# Transitional B Cells in Early Human B Cell Development – Time to Revisit the Paradigm?

**DOI:** 10.3389/fimmu.2016.00546

**Published:** 2016-12-02

**Authors:** Victoria G. Martin, Yu-Chang Bryan Wu, Catherine L. Townsend, Grace H. C. Lu, Joselli Silva O’Hare, Alexander Mozeika, Anthonius C. C. Coolen, David Kipling, Franca Fraternali, Deborah K. Dunn-Walters

**Affiliations:** ^1^Division of Infection, Immunity and Inflammatory Disease, Faculty of Life Sciences & Medicine, King’s College London, London, UK; ^2^Randall Division of Cell and Molecular Biophysics, Faculty of Life Sciences & Medicine, King’s College London, London, UK; ^3^Faculty of Health and Medical Sciences, School of Biosciences and Medicine, University of Surrey, Guildford, Surrey, UK; ^4^Faculty of Life Sciences & Medicine, Institute for Mathematical and Molecular Biomedicine, King’s College London, London, UK; ^5^Institute of Cancer and Genetics, School of Medicine, Cardiff University, Cardiff, UK

**Keywords:** bone marrow, human, B cell development, transitional, regulatory B cells

## Abstract

The B cell repertoire is generated in the adult bone marrow by an ordered series of gene rearrangement processes that result in massive diversity of immunoglobulin (Ig) genes and consequently an equally large number of potential specificities for antigen. As the process is essentially random, the cells exhibiting excess reactivity with self-antigens are generated and need to be removed from the repertoire before the cells are fully mature. Some of the cells are deleted, and some will undergo receptor editing to see if changing the light chain can rescue an autoreactive antibody. As a consequence, the binding properties of the B cell receptor are changed as development progresses through pre-B ≫ immature ≫ transitional ≫ naïve phenotypes. Using long-read, high-throughput, sequencing we have produced a unique set of sequences from these four cell types in human bone marrow and matched peripheral blood, and our results describe the effects of tolerance selection on the B cell repertoire at the Ig gene level. Most strong effects of selection are seen within the heavy chain repertoire and can be seen both in gene usage and in CDRH3 characteristics. Age-related changes are small, and only the size of the CDRH3 shows constant and significant change in these data. The paucity of significant changes in either kappa or lambda light chain repertoires implies that either the heavy chain has more influence over autoreactivity than light chain and/or that switching between kappa and lambda light chains, as opposed to switching within the light chain loci, may effect a more successful autoreactive rescue by receptor editing. Our results show that the transitional cell population contains cells other than those that are part of the pre-B ≫ immature ≫ transitional ≫ naïve development pathway, since the population often shows a repertoire that is outside the trajectory of gene loss/gain between pre-B and naïve stages.

## Introduction

B cells development starts in the bone marrow (BM), from a hematopoietic stem cell precursor, and undergoes an ordered series of differentiation steps to ultimately generate mature naïve B cells in the peripheral blood (1). As development progresses, the B cell receptor (BCR) is generated and adjusted to ensure that cells are not overly autoreactive. First, at the initial pro-B cell stage heavy chain gene recombination occurs, such that the random selection and joining of *IGHV, IGHD*, and *IHGJ* genes produces a complete heavy chain. As cells develop into pre-B cells the heavy chain is then presented on the surface of the cell, in conjunction with a surrogate light chain, so that selection of productive heavy chains can take place. Cells without a productive heavy chain gene rearrangement are removed from the repertoire, while cells containing productive heavy chains undergo a few rounds of proliferation and are designated “large” pre-B cells (2). After this point, light chain recombination of *IGK* or *IGL* genes occurs within each cell in order to produce cells with rearranged heavy (IgM) and light chain genes (3–5). Expression of the complete antibody on the surface on these immature B cells enables the first tolerance checkpoint such that some cells carrying receptors with too high an affinity for self-antigens undergo receptor editing to change the light chains (6). Lack of a functional surrogate light chain somehow interferes with this tolerance checkpoint (7). It has been shown that 55.2% (*n* = 29) of early immature B cells carried polyreactive immunoglobulin (Ig) genes, and this was reduced by receptor editing, or deletion from the repertoire, so that only 7.4% (*n* = 72) of transitional cells exiting the BM carried polyreactive antibodies (8). The term “transitional cells” was originally coined to categorize the group of early emigrant cells from the BM. These cells express IgD and CD10 alongside the IgM BCR so can be identified as IgD^+^ CD27^−^CD10^hi/+^ (9). Co-expression of high levels of CD24 and CD38 have also frequently been used to identify them, and it is important that CD27 be included if this is the case since the CD38^hi^CD24^hi^ population can contain CD27^+^ cells that may be more akin to the IgM memory populations (10). Heterogeneity has been seen within transitional cells such that T1 (CD38^+++^CD24^hi^CD10^++^IgD^lo/−^), T2 (CD38^++^CD24^hi^CD10^+^IgD^+^), and T3 (CD38^+^CD24^+^IgD^+^ABCB1^−^) subpopulations have been identified (9, 11, 12). T1 cells have been shown to be highly prone to spontaneous apoptosis and are hard to rescue even with BCR or T cell stimulation (13, 14), thereby providing another opportunity for negative selection during tolerance and removal of autoimmunity (8, 15). T2 cells were thought to be less responsive to negative selection and more responsive to antigen stimulation allowing for positive selection to occur (13, 14, 16, 17). The functional classification of CD38^hi^CD24^hi^cells as transitional cell intermediates between BM and peripheral naïve B cells in development has also been complicated by the discovery of human regulatory B cells (Bregs), which are also CD38^hi^CD24^hi^ (18).

In humans, the gradual loss of CD10, CD5, and IgM and the upregulation of CD22, CD44, CD21, and CD23 as cells progress from immature to transitional (TI to T2 to T3) to mature naïve cells, along with the generation of naïve cells from stimulated transitional cells (9, 19), lead to the current paradigm: that B cells develop from pre-B cells through immature cells in the BM to transitional cells in the periphery and then to peripheral naïve cells in a linear pathway (20).

Positive and negative selection events that occur in B cell development are expected to shape the repertoire of B cell populations in terms of V, D, J gene usage and CDRH3 properties. We have previously shown that different stages of memory B cell development have distinct repertoire characteristics (21–23). Notably, an increase in *IGHV3* family at the expense of *IGHV1* family in IgM memory cells (but not switched memory cells) (21) has been seen, and a decrease in the overall CDR3 length, which is partially (but not wholly) caused by an increase of *IGHJ4* family usage at the expense of *IGHJ6* family usage is observed in memory cells in general (21–25). The selection events that occur during central and peripheral tolerance will shape the Ig repertoire due to the removal of unwanted autoreactive cells. Comparison between passenger out-of-frame Ig genes and in-frame Ig genes in human naïve cells indicates that B cell selection has already occurred before exogenous antigen activation (26). Cloning of up to 131 Ig genes from pre-B, immature, and mature B cell subsets indicates there may be differences in CDRH3 characteristics due to negative selection processes (27). However, little information is available on the expressed Ig repertoire as a whole in the early stages of development in the human BM. Here, we have used high-throughput sequencing to define the heavy and light chain B cell repertoire in pre-B and immature cells from human BM, alongside donor-matched transitional and naïve B cells from the peripheral blood, to provide an overall picture of the consequences of early selection events on human B cell repertoire.

## Methods

### Sample Collection

Bone marrow and peripheral blood was obtained from 19 healthy adult donors (aged 24–86 years) with no known disease affecting the immune system and undergoing total hip replacement surgery at Guy’s Hospital, London, UK. The samples were collected with informed consent under the REC number 11/LO/1266.

### B Cell Isolation and Sorting

The B cells were isolated and sorted as previously published (28). Briefly, BM material was removed from the head of the femur and filtered into RPMI-1640 (Sigma-Aldrich). Bone marrow mononuclear cells (BMMCs) and peripheral blood mononuclear cells (PBMCs) were isolated using Ficoll-Paque PLUS (GE Healthcare Life Sciences) according to the manufacturer’s instructions. For the BMMCs, CD19^+^ B cells were then enriched to >98% using CD19 microbead magnetic separation (Miltenyi).

Bone marrow mononuclear cells were stained using PE anti-human Ig light chain lambda (MHL-38, BioLegend), APC anti-human Ig light chain kappa (MHK-49, BioLegend), PE/Cy7 anti-human CD38 (HIT2, BioLegend), PerCP/Cy5.5 anti-human IgD (IA6-2, BioLegend), Pacific Blue anti-human IgM (MHM-88, BioLegend), APC/Cy7 anti-human CD10 (HI10a, BioLegend), and FITC CD27 (M-T271, Miltenyi Biotec). PBMCs were stained using CD19 APC (HIB19, BD BioScience), IgD PerCP/Cy5.5 (IA6-2, BioLegend), CD27 FITC (M-T271, Miltenyi Biotec), and CD10 APC/Cy7 (HI10a, BioLegend).

**Figure 1:**
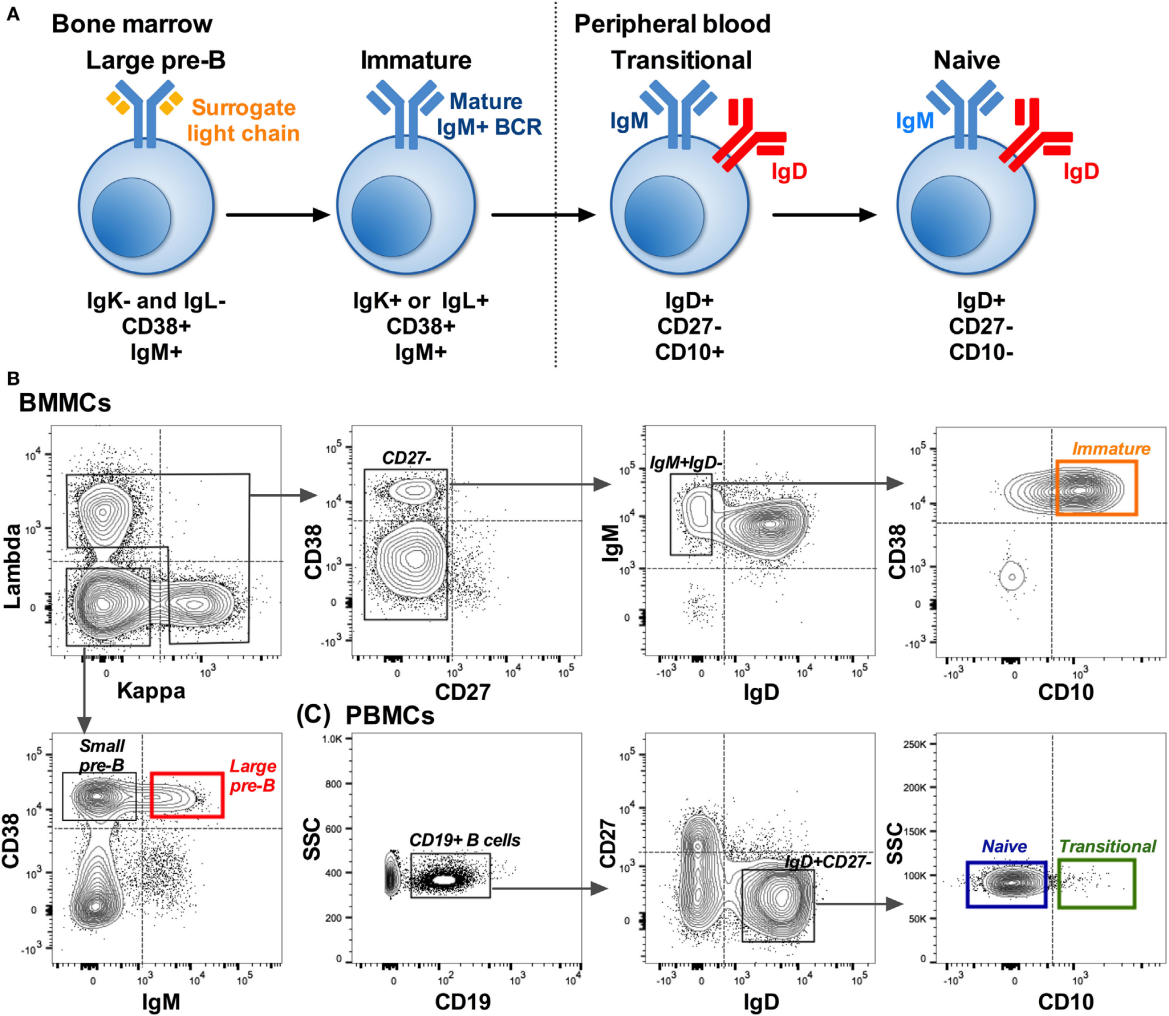
**Isolation of B cells early in development**. **(A)** B cell development pathway with phenotype used to distinguish each cell type. Starting from a CD19^+^ population: **(B)** Example showing the sorting strategy used to isolate pre-B (red: IgK^−^IgL^−^CD38^+^IgM^+^) and immature (orange: IgK^+^ or IgL^+^CD27^−^IgM^+^IgD^−^CD10^+^) B cells from bone marrow mononuclear cells (BMMCs). **(C)** Sorting strategy used to isolate transitional (green: IgD^+^CD27^−^CD10^+^) and naïve (blue: IgD^+^CD27^−^CD10^−^) cells from matched peripheral blood mononuclear cells (PBMCs). Dotted lines on the plots represent the gates based on FMO controls, and the solid lined boxes represent the gating used to collect the different subsets.

B cells were sorted into Sort Lysis Reverse Transcription (SLyRT) (21) buffer using the FACS Aria (BD BioSciences). B cells were sorted into four cell types: large pre-B (IgK^−^IgL^−^CD38^+^IgM^+^), immature (IgK^+^ or IgL^+^CD27^−^IgM^+^IgD^−^CD10^+^), transitional (IgD^+^CD27^−^CD10^+^), and naïve (IgD^+^CD27^−^CD10^−^) as shown in Figure 1. Due to the lytic (RNA stabilizing) nature of the sort buffer and the rarity of some of the cell populations, we were unable to check post-sorting purity. We set the collection gates well away from the FMO control gates as a precautionary measure (Figures 1B,C).

### High-Throughput Sequencing and Data Cleanup

High-throughput sequencing was carried out as previously described (21, 29). Briefly, reverse transcription was performed directly on the sample immediately after sorting and then a semi-nested PCR was performed, adding multiplex identifiers (MIDs) to distinguish patients (29). High-throughput sequencing was carried out using the Roche 454 GS FLX system (LGC Genomics), and data cleanup was performed as before (29). In addition, for analysis of the CDR3 peptide sequence character, the data were cleaned to remove sequences where the CDR3 was likely inaccurate as a result of sequencing error, i.e., CDR3 regions outside the normal distribution of CDR3 lengths (1–35 amino acids for heavy chain and 1–20 amino acids for light chain) and/or sequences identified by IMGT as unproductive.

V(D)J gene assignment was carried out using IMGT/HighV-QUEST (30, 31). The physicochemical properties of the CDR3 amino acid sequences were calculated using the R package Peptides (32, 33), and clustering analysis of the Ig gene sequences was carried out using Levenstein distance on the CDR3 regions using R scripts available on our website (34).

As all of the repertoires were antigen-naïve, then true clonal expansions would be negligible. Therefore, in order to remove biases caused by PCR amplification, only unique gene rearrangements were used for this analysis. Where the clustering identified more than one related sequence, a modal sequence was used to represent the gene rearrangement. The data were stored in CSV files, and data analysis was performed using Microsoft Excel, GraphPad Prism, and R.

## Analysis and Statistics

### Frequency of Gene Usage in the Repertoire

The frequency of each gene (both at the individual gene and at the gene family level) observed in the data was calculated for each cell subset from each donor. The frequency (in percentage) of each VDJ family combination (heavy chain) or VJ family combination (light chain) was also calculated for each cell subset from each donor. The mean values of gene combination frequencies were calculated for each cell subset, and 3D bubble plots were created using the R package *plot3D* (35). Statistical analysis (Mann–Whitney, Wilcoxon test, and ANOVA, with post-test analysis where appropriate) was performed using R or GraphPad Prism.

### Physicochemical Properties of CDR3 Regions

The physicochemical properties of CDR3 regions at heavy and light chains were compared between different cell types. These properties consisted of length, hydrophobicity indicated by GRAVY index (36), Boman index (37), molecular weight (Mr), isoelectric point (pI) (38), aliphatic index (39), frequency of amino acid classes in the CDR3 region, and Kidera factors (40). The R package *lem4* (41) was used for fitting and analyzing the mixed model of our data, describing the fixed-effect (cell types) and the random-effect (patients) in a linear predictor expression. The likelihood ratio test was calculated with the statistical method ANOVA to estimate the statistical significance between populations, i.e., a pair of cell subsets.

### Clustering and Principal Component Analysis

Principal component analysis (PCA) and clustering, using Minkowski distance, were applied to the Kidera factors and gene usage frequencies from the CDR3 data as follows. First, the mean values of the Kidera factors and gene usage frequencies were computed for each donor. Second, the mean values and frequencies of all donors were grouped and then analysed by PCA and clustering.

Principal component analysis was performed using the *prcomp* function in R. The Minkowski distances (with power of 4) were calculated using *dist(method* = “*minkowski”)* function in R based on all CDR3 properties. The distances were then plotted with dendrograms (trees) using the *dendrapply* function in R.

Randomise datasets were generated by randomly shuffling the sequences across four cell subpopulations. PCA analysis was then performed to be compared with the original dataset in order to show that our observations of differences between cell subpopulations were not random events.

### Mass Cytometry

Peripheral blood mononuclear cells were stained with FITC anti-human CD14 and APC anti-human CD3 (clone M5E2 and UCHT1, respectively), and a population of enriched B cells (CD3^−^CD14^−^) was collected into 50% FCS (Biosera) and 50% RPMI-1640 (Gibco). The CD3^−^CD14^−^ enriched B cells were labeled with a rhodium intercalator (Rh103, DVS Sciences) followed by intracellular and extracellular staining with a panel of 30 different metal-tagged antibodies (DVS Sciences, BD BioSciences, and BioLegend). Cells were fixed, iridium stained (Ir193, DVS Sciences), and normalization beads (DVS Sciences) were added before analysis on the mass cytometry system (DVS Sciences). Between 1 and 5 × 10^5^ stained cells were analysed per sample.

Data were normalized and files were concatenated and cleaned up to remove debris (by gating on cell length and DNA^+^ cells), to exclude normalization beads (Ce140^−^ cells), to positively select intact cells (Ir191^+^Ir193^+^), to positively select live cells (Rh103^−^Ir193^+^), and to identify CD19^+^ and/or CD20^+^ B cells. CD38^hi^CD24^hi^ B cells were identified and exported as a new group prior to performing SPADE (Spanning-tree Progression Analysis of Density-normalized Event) analysis (42). SPADE analysis groups cells into “nodes” based on the expression of all 30 markers to produce a two-dimensional tree. Using a color coded expression scale, the nodes in the tree were manually grouped into larger “bubbles” to collect together nodes, and therefore cells, which had similar expression, i.e., all those with high IgM expression were grouped together in one bubble.

## Results

### Heavy Chain Gene Family Usage Distinguishes Cell Types

Pre-B (large pre-B) and immature B cells, from BM samples, and matched transitional and naïve B cells, from PB samples, were sorted (Figures 1B,C) prior to high-throughput sequencing using an IgM-specific constant region primer. Both the heavy and light chain (kappa and lambda) Ig genes were amplified with a total of 96,593 heavy and 49,101 light chain sequences generated after initial data cleanup. These B cell populations are all thought to be exogenous antigen-naïve and therefore will not have been activated to undergo somatic hypermutation and expansion. We do not see evidence of somatic hypermutation in the gene sequences (data not shown), and therefore, we have assumed that any sequences with the same CDR3 region arise from PCR duplication. Therefore, only one example sequence of any unique gene rearrangement was used in this analysis, resulting in 39,577 heavy chain and 42,542 light chain sequences grouped by donor and cell type. Sequencing error does not substantially affect the assignment of germline Ig genes to the sequences; however, for the CDR3 peptide analysis we further removed sequences where the peptide sequence may be inaccurate due to sequencing error. This resulted in 29,074 heavy chain and 29,128 light chain sequences (Supplementary Tables). Sequences can be accessed on the National Center for Biotechnology Information’s Sequence Read Archive in raw format (BioProject: PRJNA39946; Sequence Read Archive accession: SRP081849) or in processed format with metadata at www.bcell.org.uk.

### Gene Family Repertoire Can Distinguish Early Human B Cell Subsets

**Figure 2:**
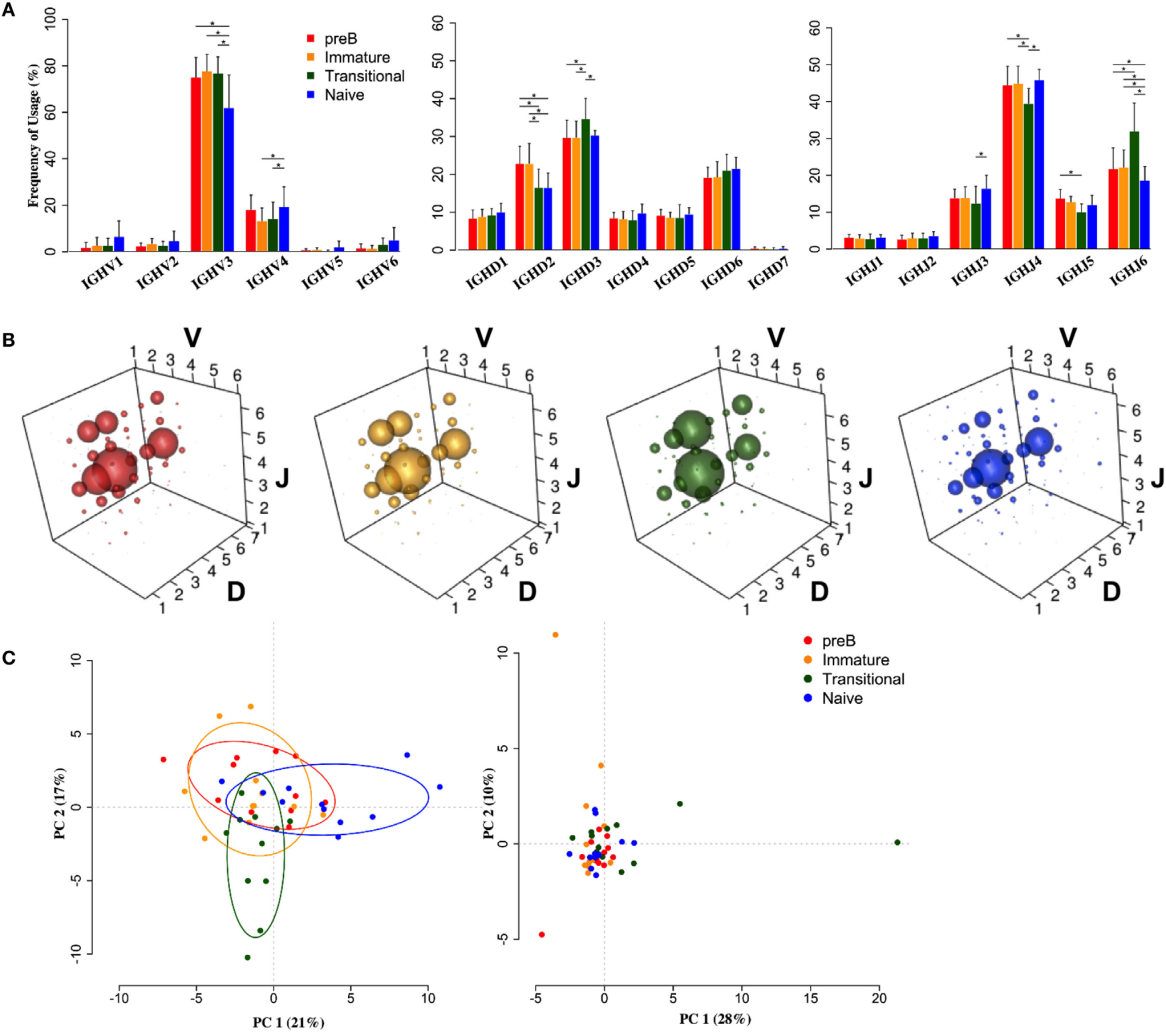
**Heavy chain VDJ gene family usage distinguishes cell types**. **(A)** Mean frequency histograms of individual V, D, and J family usage for the heavy chain gene families of Pre-B (red), immature (yellow), transitional (green), and naïve (blue) cells (*p < 0.05 by two way ANOVA with multiple analysis correction. Error bars are SEM). **(B)** VDJ family combination usage in the different cell types. The size of a bubble represents the mean frequency of that VDJ combination. **(C)** Transitional and naïve cells show difference in VDJ family usage by principle component analysis (PCA) (left) compared to a randomise data set (right).

Heavy chain V, D, and J family usage did not show any significant differences in repertoire between pre-B and immature cells from the BM. There were, however, significant differences between these BM cells and the peripheral transitional and naïve cells (Figure 2). *IGHV3* family genes are the most predominant genes in the human peripheral repertoire. It was interesting that in the BM this was particularly the case, with *IGHV3* cells actually being removed from the repertoire during B cell maturation: there is a highly significant >13% decrease in the use of *IGHV3* family genes in naïve cells with small increases in all other families to compensate (Figure 2A). Naïve cells also showed a significantly decreased use of *IGHJ6* and, together with transitional cells, a >6% reduction in use of *IGHD2* family genes.

Since we had expected that peripheral transitional cells would fall between immature BM cells and peripheral naïve cells in the development pathway, and that any changes in repertoire we saw would reflect this, we were surprised to see that this was not always the case. There was a significant 5% increased frequency of *IGHD3* family usage in transitional cells compared to all other cell types. Furthermore, there was a significant >9% increase in *IGHJ6* usage, compensated for by decreases in *IGHJ3*, 4, and 5 usages, in transitional cells compared to all other cell types. This is reflected in the different size of bubble V3D3J6 in the bubble plots (Figure 2B). The different repertoire of transitional and naïve cells compared to the BM cells (*p* < 0.05, Wilcoxon) and compared to each other (*p* < 0.001, Wilcoxon) is illustrated by a PCA analysis of gene family usage (Figure 2C).

### Light Chain Repertoire Is Less Variable

**Figure 3:**
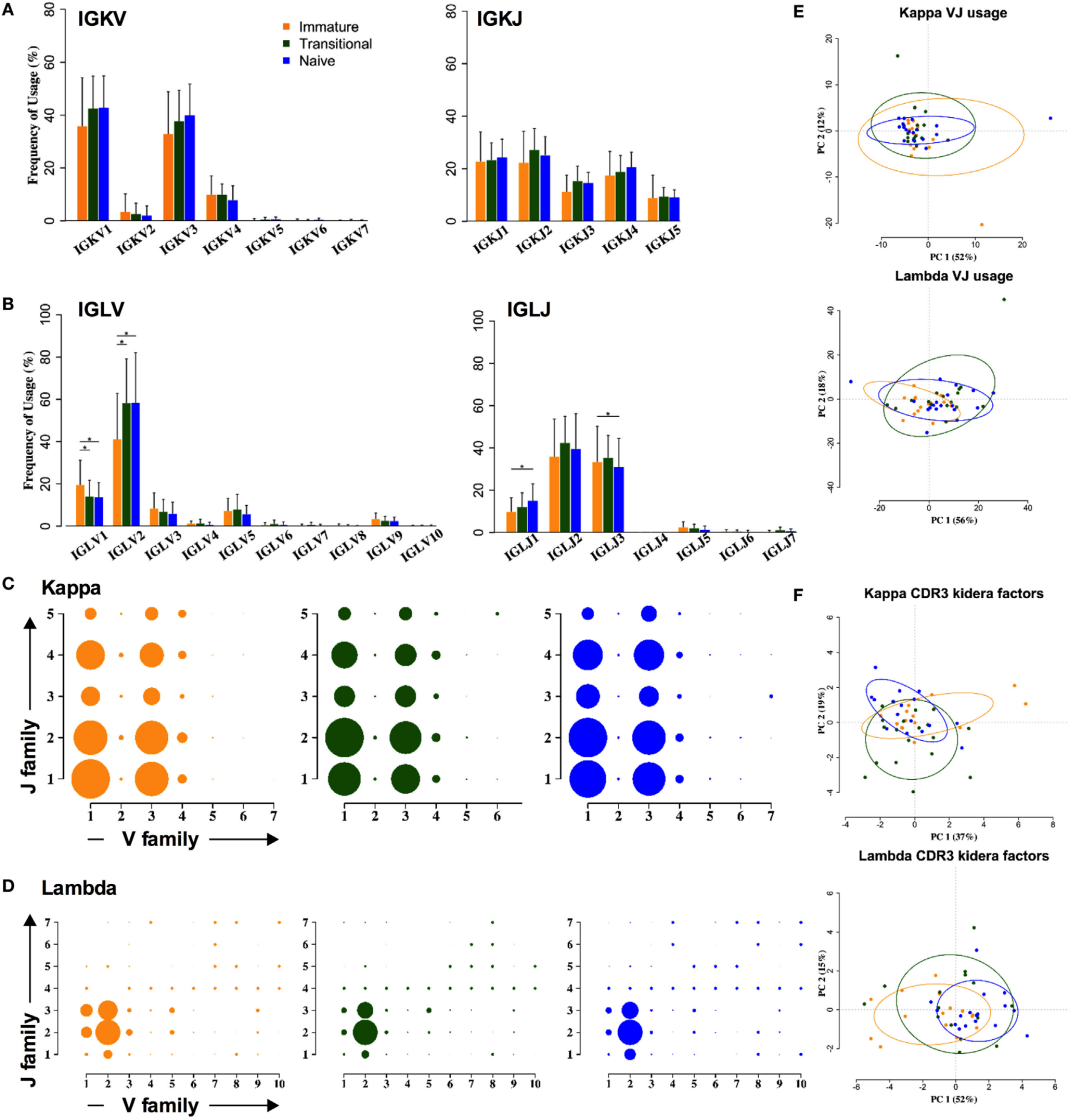
**Light chain gene usage and CDR3 properties cannot distinguish between cell types**. **(A,B)** V and J family usage for kappa **(A)** and lambda **(B)** light chain gene families between immature (yellow), transitional (green), and naïve cell types (*p < 0.05 by two way ANOVA with multiple analysis correction. Error bars are SEM). **(C,D)** Light chain VJ usage for kappa **(C)** and lambda **(D)** light chains in immature (yellow), transitional (green), and naïve (blue) B cells. The size of a circle indicates the relative mean frequency of the VJ combination. **(E,F)** Principle component analysis (PCA) of VJ usage **(E)** and Kidera factors **(F)** in three different cell types for kappa (top) and lambda (bottom).

In contrast to the heavy chain repertoire, the light chain gene family repertoire does not distinguish between cell types. There are no significant changes in kappa family usage (Figure 3A). Some differences were seen in lambda families (Figure 3B). The *IGLV2* family usage is significantly increased by 10–15%, at the expense of all other families, and *IGLJ1* family usage is significantly increased by 2–5%, at the expense of *IGLJ3*. As a result of this, an ANOVA analysis of the combinatorial lambda family repertoire showed a significant difference between the immature and the transitional and naïve stages of development (*p* < 0.001) (Figures 3C,D). However, clustering by PCA showed that any differences in light chain VJ gene usage were not strong enough to be able to distinguish between the different cell types (Figure 3E). Nor were there any obvious differences between the different cell types in lambda CDR3 amino acid sequence, since PCA of the Kidera factors to assess the physicochemical character of the CDR3 did not distinguish between the groups (Figure 3F).

### Selection of Individual *IGH* Genes in Early Development

**Figure 4:**
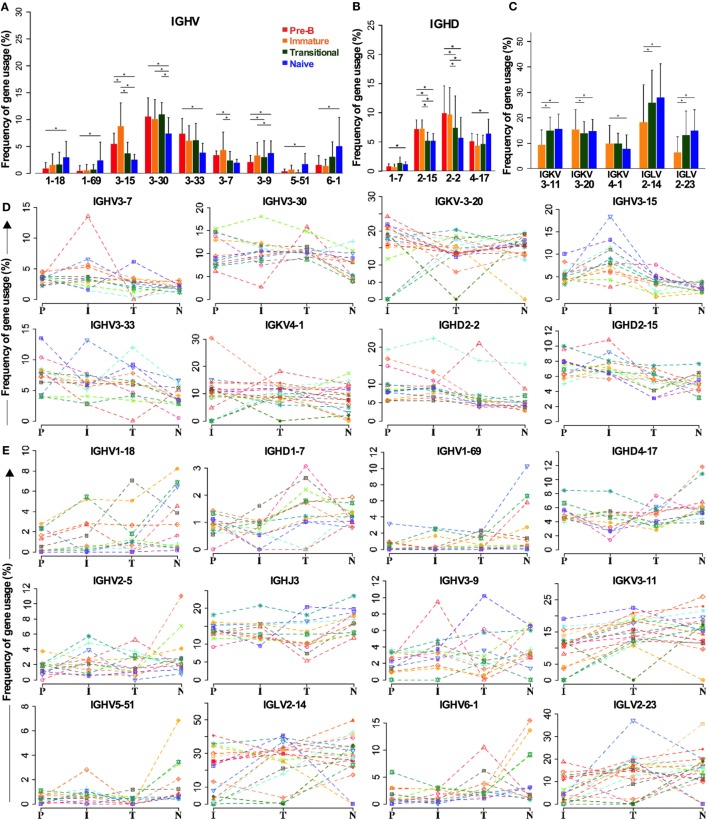
**Individual genes can be favored or disfavored as B cells mature**. **(A–C)** Frequency of IGHV **(A)** and IGHD **(B)** gene usage in heavy chain and IGKV and IGLV usage in light chains **(C)** of different cell types are compared (*p < 0.05 by two way ANOVA with multiple analysis correction. Error bars are SEM). **(D,E)** The frequency for each cell type in each individual donor is shown for genes that are decreased during selection **(D)** and those that are increased **(E)**.

As the above analysis of gene family repertoire indicated that there were repertoire changes between cell types, we analyzed all the genes individually to check if we had missed any significant gene selection due to the averaging effect of looking at the family level (Figure 4). Not all the *IGHV3* family genes are decreased in naïve cells compared to BM cells. While there are significant decreases in *IGHV3-15, IGHV3-30*, and *IGHV3-33* in particular, *IGHV3-9* is actually increased (Figure 4A). Other notable increases occur in the two main *IGHV1* family genes: *IGHV1-18* and *IGHV1-69*, and in the *IGHV6* gene. The *IGHD2* family decreases are contributed by *IGHD2-15* and *IGHD2-2*, and while the compensatory increase in other IGHD genes seemed unremarkable across the board, *IGHD1-7* and *IGHD4-17* did show significant differences (Figure 4B). In spite of the significant change in *IGHD3* family use in transitional cells, this did not show up at the individual gene level, implying that the increase occurs throughout the *IGHD* gene family. Despite the lack of significant changes in *IGK* family repertoire, there was a small (~3.8%) but significant increase in *IGKV3-11* gene use in naïve cells compared to immature cells. This appeared to be at the expense of small (<3%) decreases in *IGKV3-20* and *IGKV4-1* genes. The increase in *IGLV2* family during development seemed to be mainly due to significant increases of 12.8 and 7% in *IGL2-14* and *IGL2-23*, respectively (Figure 4C).

There is a certain amount of interindividual variation that occurs in these analyses, but the trends for selection of these genes in the repertoire are consistent, as illustrated in Figure 4, where the individual donors are shown separately for genes that are removed from the repertoire (Figure 4D) or that are increased in the repertoire (Figure 4E) during early development.

### Heavy Chain CDR3 Properties Are Also Strongly Selected

**Figure 5:**
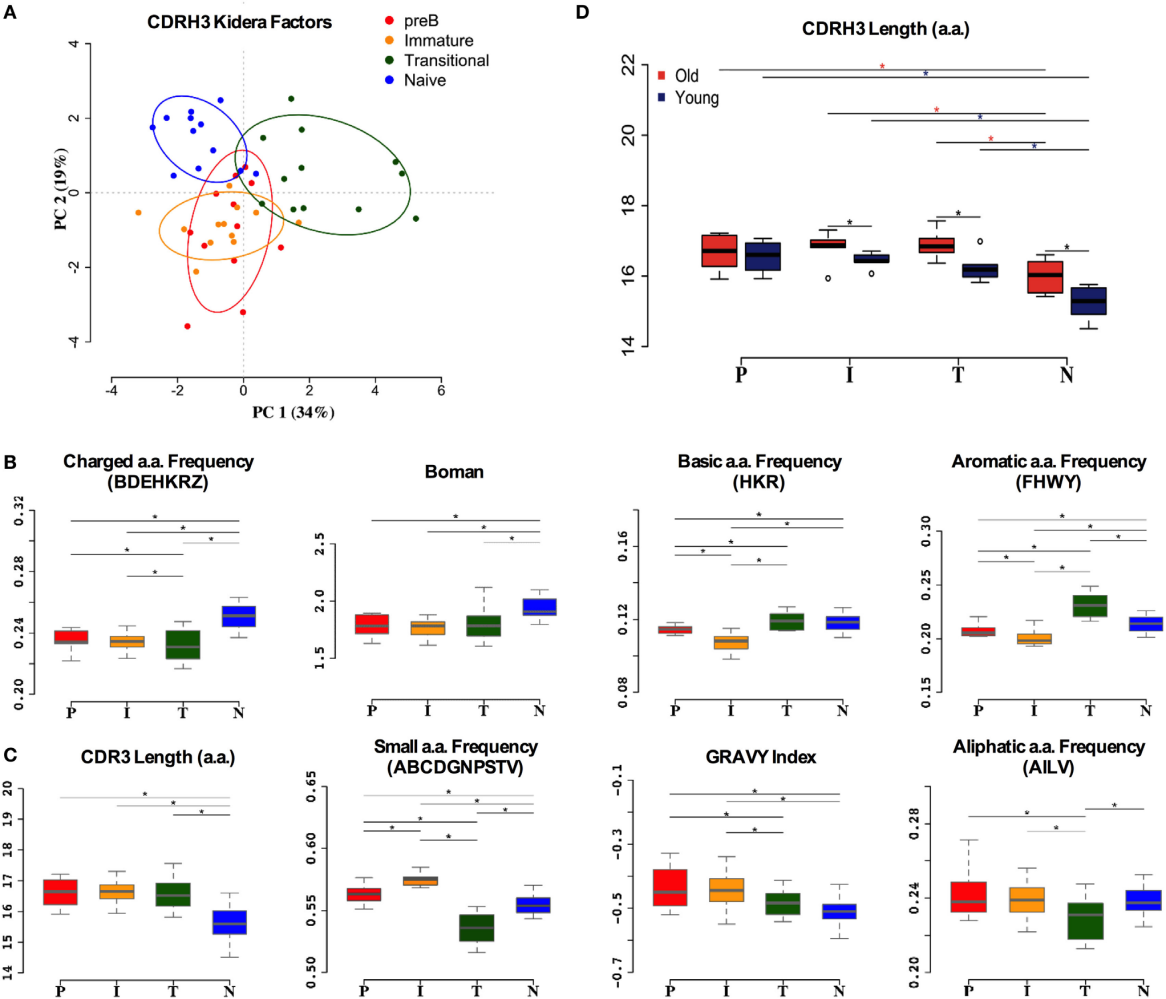
**Heavy chain CDR3 characteristics distinguish between cell types**. **(A)** Distinction between the different cell types by Kidera factors as illustrated by principal component analysis (PCA). Distribution of CDRH3 physicochemical properties that have an increased trend from pre-B (P), immature (I), transitional (T) to naïve (N) cells **(B)**, and a decrease in naïve cells compared to pre-B cells **(C)** (*p < 0.05 ANOVA). **(D)** The heavy chain CDR3 length in all cell types in young and old donors (young donors: 18–50 years; old donors: over 65 years) (*p < 0.05 ANOVA). Values on the *y* axis of **(B–D)** are as per the individual graph titles.

Although much of the CDR3 region is comprised of contributions from the individual *IGHV, IGHD*, and *IGHJ* genes, reflecting some of the repertoire selection effects that are captured in the analysis above, the actual amino acid sequences encoded by CDR3 varies tremendously even within the same VDJ combinations. In addition to the direct effects of endonuclease action on the genes, and N region insertion by terminal deoxynucleotidyl transferase, the reading frame of the *IGHD* region can also vary. Since the CDR3 region encodes a crucial part of the antibody binding site, and key functional aspects of its structure are dependent on the primary sequence (43), we also analyzed the biophysical characteristics of the CDR3 amino acid sequence. Initially we used Kidera factors, which are a set of 10 orthogonal factors that encapsulate information from ~140 different measurable biophysical characteristics of peptides. The data from PCA analysis of the CDR3 Kidera factors are in accordance with that for the VDJ gene analysis, showing that the characteristics of pre-B and immature cells are found in overlapping clusters (Figure 5A). Naïve cells and transitional cells, however, form separate yet non-overlapping clusters. The data from heavy chain CDR3 Kidera analysis separate the groups of cells better than the gene usage data, with 30% of the data contributing to PC1. To elucidate which characteristics were mainly responsible for the differences, we analyzed some of the most common ones individually. The numbers of charged, basic, and aromatic amino acids in each sequence, and the sequence Boman index, were significantly increased in naïve cells compared to pre-B cells (Figure 5B). Conversely, the number of small amino acids per sequence, the hydrophobicity (GRAVY index), aliphatic index, and overall length of sequence were all disfavored characteristics that were removed from the repertoire during development (Figure 5C). Interestingly, the selection on the size of CDR3 region did not seem as strong in the older donors as it did in the younger ones (Figure 5D).

### Human Transitional Cells Are Not Just Precursors to Naïve Cells

**Figure 6:**
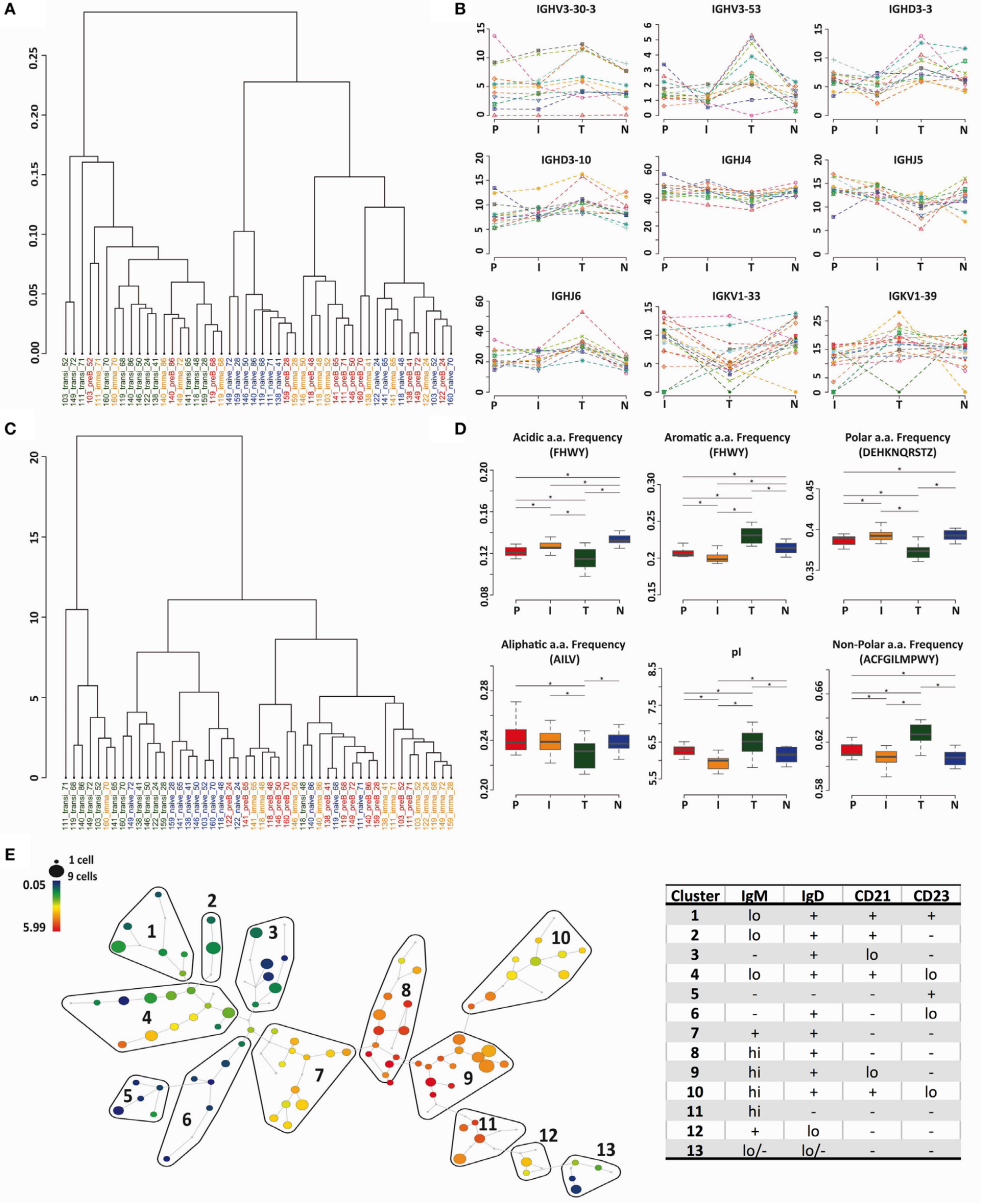
**Transitional cells have a unique heavy chain immunoglobulin repertoire**. **(A,C)** Minkowski distance clustering analysis of heavy chain VDJ family usage **(A)** and CDRH3 Kidera factors for pre-B (P) immature (I), transitional (T), and naïve (N) cells in each donor **(C)**. **(B)** The frequency of gene use (%) for different cell types in each individual donor for genes that have a distinctive distribution in transitional cells. **(D)** CDRH3 physicochemical properties in different cell types for properties that have distinctive distributions in transitional cells (*p < 0.05 ANOVA). Values on the *y* axis are as per the individual graph titles. **(E)** High-dimensional clustering of CD24^hi^CD38^hi^ transitional B cells indicates heterogeneity within the transitional population with respect to IgM expression, illustrated as a SPADE plot. Populations numbered 1–13 have been grouped according to the expression of IgM, IgD, CD21, and CD23, as shown in Figure S1 in Supplementary Material.

The heavy chain gene and CDR3 PCA analysis (Figures 2C and 5A) indicated that transitional cells, in addition to being distinctive from pre-B cells and immature cells, also had a different repertoire to naïve cells. We used cluster analysis (based on Minkowski distances) to investigate the relationships further, which confirmed, by both VDJ usage (Figure 6A) and Kidera factors (Figure 6C), that transitional cells have a different repertoire to the other cell types. Naïve cells formed a sub-branch of the cluster containing pre-B and immature cells suggesting that the naïve repertoire is more similar to the BM cells than to the transitional cells. Clear examples of individual genes where the usage in transitional cells differs from the rest of the cells can be seen in Figure 6B, and biophysical characteristics showing the significantly different character of the heavy chain CDR3 in transitional cells are shown in Figure 6D. Since this subset of cells has been reported to contain Bregs, as well as being the precursor to naïve B cells, we investigated the heterogeneity of the population by mass cytometric analysis of surface markers. Although the population is small, it does appear to contain a number of different potential subpopulations, as illustrated by the IgM SPADE plot in Figure 6E.

## Discussion

The lack of difference between the heavy chain repertoire in pre-B and immature B cells implies that there is very little selective pressure at this developmental stage, which is in agreement with current thinking on the tolerance checkpoints (44). As expected, we do see a major difference between immature BM B cells and the transitional and naïve mature peripheral B cells, where we would expect the repertoire to reflect the changes incurred as a result of the post-immature selective processes that can remove up to 50% of the repertoire (8). There is a wealth of literature on the heavy chain gene usage in different conditions, and both negative and positive associations have been made for various genes. For example, the common *IGHV1* family genes *IGHV1-18* and *IGHV1-69* have been associated with responses to viral infections as well as with stereotypical receptors in CLL. It is interesting that these two genes increase, and a number of *IGHV3* family genes decrease, since this recapitulates the change in repertoire between naïve and switched memory repertoire (21). Indeed, the relative use of *IGHV1* and *IGHV3* genes seems to be a marker that distinguished between a number of different B cell types (25). Furthermore, the significant changes in CDRH3 are to be expected from a selected population, since this forms the most important part of the antibody-binding site in all except the smallest CDRH3 regions. What was particularly striking from these data was that the selection in CDRH3 appeared to change with age even at this early stage in development, particularly in the length of the CDRH3 region. We, and others, have previously noted that shorter CDRH3 regions are selected upon exogenous antigen selection (21, 28, 45), and that older people have longer CDRH3 regions than in the young when measured in peripheral blood IgM-expressing cells. These data show that a longer CDRH3 exists in B cells even before exogenous antigen stimulation so is likely a result of changes in BM tolerance selection rather than any exogenous antigen selection of IgM memory cells.

Receptor editing to rescue potentially autoreactive B cells can occur after the immature B cell stage once the light chain has been co-expressed. The light chain loci continues its rearrangement to form a new gene. The kappa light chain locus rearranges before the lambda locus and has the potential to rearrange a number of times. However, at some point, the kappa locus would run out of genes to rearrange, or the kappa deleting element would be used, in which case then the lambda locus would start rearrangement (3, 5). With this in mind, the paucity of differences in light chain repertoire between immature, transitional, and naïve cells is quite surprising. The kappa repertoire in particular does not change much, possibly indicating that that the ability of different kappa genes to rescue a potentially autoreactive heavy chain gene does not vary much. Only *IGKV3-20* and *IGKV4-1* show a significant decrease in use (Figure 4C), implying a potential contribution to autoreactive BCR. Indeed, *IGKV4-1* has previously been shown to be overrepresented in systemic lupus erythromatosus, celiac disease, and type 1 diabetes (46, 47), and we have also shown that its actual expression in the peripheral repertoire is significantly lower than its frequency of rearrangement in the genomic DNA (48). *IGKV3-11* may possibly be a rescue gene, showing a significant increase in use, and our previous analysis also showed an increase in expression of this gene in the expressed repertoire compared to its expected frequency of rearrangement (48). Two *IGLV2* lambda genes were noted as being increased within the lambda repertoire, presumably in preference to the *IGLV1* family genes that showed a slight decrease. Not much is known about the potential significance of lambda light chain genes, although it has been reported that POEMS syndrome of neuropathy is associated with monoclonal expansions of *IGLV1* family plasma cells (49). It has been reported that lambda light chains have a good potential for rescuing autoreactive B cells (50). Since the primer sets we used for these experiments amplified the kappa and lambda light chains separately, we cannot comment on any changes in kappa/lambda ratio between immature and later B cells. Given the inability of the light chain repertoire characteristics to distinguish between the different cell types, as shown by the PCA of Figures 3E,F, it is possible that any light chain-mediated autoreactive rescue would be more likely to be performed by a switch from kappa to lambda than by a switch within the loci. Alternatively, the lack of cell type-distinguishing features in the light chain repertoire could mean that the central selection events are mainly driven by heavy chain-encoded binding specificities. The selection in heavy chain but not light chain also implies that the heavy–light chain pairing is mostly random, since if the pairing had biases then the same selection effects would appear in both chains. This is in agreement with previous data where a large number of paired heavy and light chain rearrangements were sequenced (51, 52). It has been previously reported that a particular CDRH3 stereotype on a *IGHV1-69* background might be associated with a particular light chain gene, but this was on a small sample size (*n* = 66) of selected CLL sequences (53), and the data here represent a much larger diversity in a normal unselected population of cells.

What we had not expected to see in these data was the large difference between transitional and naïve B cells, which does not seem in accord with an immature–transitional–naïve pathway of development. One assumes that processes in nature have evolved to require minimum energy or resource, and if this is the case, then any change in repertoire between creation (pre-B cells) and end point (naïve B cells) would be in a single linear direction. The actual cell–cell differences may vary depending on which point the selection pressure were applied, but one would not expect to see a change in direction of increase/decrease one way, followed by a change in direction back again, half way through a development pathway, i.e., for a gene that was being removed from the repertoire through the development pathway we would expect the percentage representation in the repertoire to be pre-B > immature > transitional > naïve. In actual fact, for some genes, we see varying patterns such as transitional > (pre-B = immature) > naïve. For this reason, and in the light of results exemplified by use of *IGHV3-53* or use of non-polar CDR3 amino acids (Figures 6B,D), we assume that a large proportion of the cells in our transitional subset are *not* intermediates between BM immature and peripheral naïve B cells. We sorted our CD19^+^IgD^+^CD10^hi^CD27^−^ cells, based on the previous information that CD10, CD24, and CD38 decrease as cells develop from immature to naïve. This information had been obtained by studying the reconstitution of different phenotypic subsets after B cell depletion (9). There are three subsets of mature non-memory B cells by the expression of CD10 (high, medium, and low) that have parallels in the differing strengths of CD24^hi^CD38^hi^ expression in humans. These distinctions were first described in mice as T1, T2, and T3 subsets, and this nomenclature has been carried over into human studies (54). The transitional cell subset in humans has been shown to contain B cells with regulatory activity after stimulation *in vitro* (55) and have also been shown to contain cells with different homing integrins (56). It is clear from our high-dimensional phenotyping in Figure 6 that the population can be quite heterogeneous. Since the FACS gates that we used were quite stringent, we skewed our cells toward the equivalent of the mouse T1 population, which may be less diverse and less representative of that portion of cells that are precursors to naïve cells. In this context, it is interesting that a prior comparison of human T1 and T2 cells also showed a difference in *IGHJ6* usage (11). Without the immature B cell repertoire to give this context this could be interpreted as *IGHJ6* being removed gradually from the repertoire. However, in the light of the fact that our transitional cells have higher *IGHJ6* than either immature cells or naïve cells then this is unlikely. In reality, this CD10 very high population has a very distinctive repertoire in many other respects also, and therefore likely has a completely different function. Whether this would be the Breg subset or not would require further investigation in the future.

In summary, we have shown that there are strong selective influences over the B cell repertoire in early B cell development, and we can identify genes and characteristics that are likely to be detrimental by the fact that they disappear from the repertoire in development. The selection effects are mainly on the heavy chain rather than the light chain genes. This is surprising considering the role that receptor editing is thought to play in central tolerance and may mean that either the heavy chain plays a dominant role in receptor specificity or that switching between kappa and lambda is the chief mode of receptor editing. An unexpected finding was that the transitional subset of cells with the highest level of CD10 expression may not really be a transitional stage between immature and naïve B cells, and further work will be required to determine whether these represent the Bregs.

## Ethics Statement

This study was carried out in accordance with the recommendations of the NRES committee London – Bromley 11/LO/1266. Patients were approached minimum 3 weeks prior to their operation at their pre-operative rehabilitation meeting. A short presentation on the project was given, and they had at least an hour to read the patient information sheet. Consent was taken at the meeting, and the patients were told they could withdraw at any time. All forms were as approved by the REC, and all samples were kept anonymised.

## Author Contributions

DK, DD-W, VM, GL, and CT analyzed data; Y-CW, JH, and VM performed experiments; AM, FF, and AC advised on statistics and bioinformatics methods; FF, AC, and DD-W directed the data analysis. DK, DD-W, VM, GL, CT, and Y-CW wrote the paper. DD-W designed the experiments and directed the project.

## Conflict of Interest Statement

The authors declare that the research was conducted in the absence of any commercial or financial relationships that could be construed as a potential conflict of interest.

## Supplementary Material

Figure S1 **High-dimensional clustering of CD24^hi^CD38^hi^ transitional B cells indicates heterogeneity within the transitional population with respect to IgD, CD21, and CD23 expression, illustrated as a SPADE plot**. Populations numbered 1–13 have been grouped according to expression of IgM, IgD, CD21, and CD23; see Figure 6E for a tabulated summary.
