# Supplementary material for: Transitional B Cells in Early Human B Cell Development – Time to Revisit the Paradigm?
Source: Front Immunol. 2016 Dec 2;7:546. doi: 10.3389/fimmu.2016.00546 (PMC5133252; doi:10.3389/fimmu.2016.00546)
Supplement: Supplementary file 1 [file Data_Sheet_1.PDF]

	Heavy chain	Light chain
**Sequencing data**	96,593	49,101
**After removing clonal expansion**	39,577	42,542
**After removing sequencing error entries**	29,074	29,128

After removing clonal expansion-Heavy chain

**patients**	**103**	**111**	**118**	**119**	**122**	**138**	**140**	**141**	**146**	**149**	**159**	**160**	**Total**
**preB**	87	620	2145	2959	1290	2627	730	721	3824	1118	829	89	17039
**Immature**	364	74	476	1697	284	76	435	1483	675	1132	1879	189	8764
**Transitional**	65	19	293	905	343	195	340	975	463	178	502	98	4376
**Naïve**	1606	1106	647	367	259	496	571	498	245	146	349	3107	9397
**Total**	2122	1819	3561	5928	2176	3394	2076	3677	5207	2574	3559	3483	39576

After removing sequencing error entries - Heavy chain

**patients**	**103**	**111**	**118**	**119**	**122**	**138**	**140**	**141**	**146**	**149**	**159**	**160**	**Total**
**preB**	69	499	1312	1947	935	2047	579	555	2885	889	633	62	12412
**Immature**	281	59	392	1306	220	54	309	1083	507	816	1342	149	6518
**Transitional**	56	13	192	680	274	154	269	577	351	126	380	81	3153
**Naïve**	1243	811	428	245	202	379	434	359	172	105	260	2353	6991
**Total**	1649	1382	2324	4178	1631	2634	1591	2574	3915	1936	2615	2645	29074

After removing clonal expansion-Light chain

**patients**	**103**	**105**	**107**	**111**	**118**	**119**	**120**	**122**	**126**	**128**	**132**	**138**	**140**	**141**	**146**	**149**	**159**	**160**	**162**	**Total**
**Immature**	531	226	0	257	808	3699	0	1670	165	208	0	269	893	3330	1459	1508	1620	487	0	17130
**Transitional**	18	265	2125	216	335	686	1395	391	894	580	1262	254	435	700	588	0	553	200	1130	12027
**Naïve**	857	718	315	1322	339	488	588	550	2532	0	776	954	1128	541	167	413	661	786	250	13385
**Total**	1406	1209	2440	1795	1482	4873	1983	2611	3591	788	2038	1477	2456	4571	2214	1921	2834	1473	1380	42542

After removing sequencing error entries - Light chain

**patients**	**103**	**105**	**107**	**111**	**118**	**119**	**120**	**122**	**126**	**128**	**132**	**138**	**140**	**141**	**146**	**149**	**159**	**160**	**162**	**Total**
**Immature**	366	130	0	154	544	2430	0	1193	94	123	0	153	587	2233	941	936	1031	284	0	11199
**Transitional**	10	202	1521	155	245	531	923	235	467	334	766	205	291	500	387	0	360	155	784	8071
**Naïve**	649	582	268	982	246	370	476	374	1690	0	589	692	801	382	114	297	504	651	191	9858
**Total**	1025	914	1789	1291	1035	3331	1399	1802	2251	457	1355	1050	1679	3115	1442	1233	1895	1090	975	29128
