# Supplementary figures and images for: Transitional B Cells in Early Human B Cell Development – Time to Revisit the Paradigm?
Source: Front Immunol. 2016 Dec 2;7:546. doi: 10.3389/fimmu.2016.00546 (PMC5133252; doi:10.3389/fimmu.2016.00546)

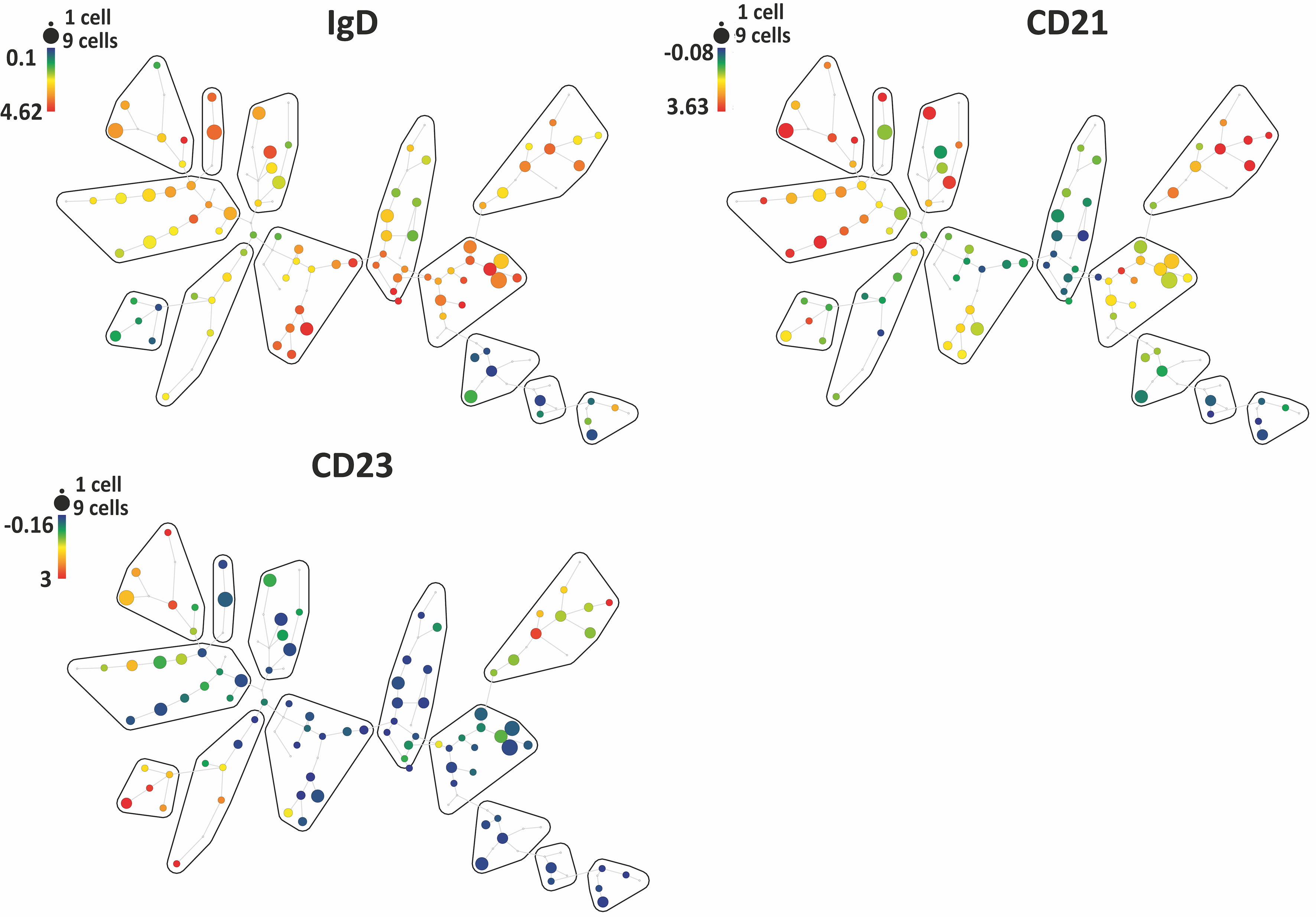

Supplement: Figure S1 — High-dimensional clustering of CD24hiCD38hi transitional B cells indicates heterogeneity within the transitional population with respect to IgD, CD21, and CD23 expression, illustrated as a SPADE plot. Populations numbered 1–13 have been grouped according to expression of IgM, IgD, CD21, and CD23; see Figure 6E for a tabulated summary. [file Image_1.tif]
